# Steric Clash in the SET Domain of Histone Methyltransferase NSD1 as a Cause of Sotos Syndrome and Its Genetic Heterogeneity in a Brazilian Cohort

**DOI:** 10.3390/genes7110096

**Published:** 2016-11-09

**Authors:** Kyungsoo Ha, Priya Anand, Jennifer A. Lee, Julie R. Jones, Chong Ae Kim, Debora Romeo Bertola, Jonathan D. J. Labonne, Lawrence C. Layman, Wolfgang Wenzel, Hyung-Goo Kim

**Affiliations:** 1 Section of Reproductive Endocrinology, Infertility & Genetics, Department of Obstetrics and Gynecology, Department of Neuroscience and Regenerative Medicine, Medical College of Georgia, Augusta University, 1120 15th Street, Augusta, GA 30912, USA; kyungsooha1@gmail.com (K.H.); molgenetics_and_epigenetics@hotmail.com (J.D.J.L.); lalayman@augusta.edu (L.C.L.); 2 New drug development center, Osong Medical Innovation Foundation, 28160 Cheongju, Korea; 3 Institute of Nanotechnology (INT), Karlsruhe Institute of Technology (KIT), 76344 Karlsruhe, Germany; priya.anand@kit.edu (P.A.); wolfgang.wenzel@kit.edu (W.W.); 4 Molecular Diagnostic Laboratory, Greenwood Genetic Center, Greenwood, SC 29646, USA; jalee@ggc.org (J.A.L.); juliejones@ggc.org (J.R.J.); 5 Unit of Clinical Genetics, Instituto da Criança, FMUSP, São Paulo 05403-000, Brazil; chong.kim@hc.fm.usp.br (C.A.K.); debora.bertola@hc.fm.usp.br (D.R.B.); 6 Neuroscience Program, Medical College of Georgia, Augusta University, 1120 15th Street, Augusta, GA 30912, USA

**Keywords:** Sotos syndrome, NSD1, SET domain, histone methyltransferase, intellectual disability

## Abstract

Most histone methyltransferases (HMTase) harbor a predicted Su(var)3–9, Enhancer-of-zeste, Trithorax (SET) domain, which transfers a methyl group to a lysine residue in their substrates. Mutations of the SET domains were reported to cause intellectual disability syndromes such as Sotos, Weaver, or Kabuki syndromes. Sotos syndrome is an overgrowth syndrome with intellectual disability caused by haploinsufficiency of the nuclear receptor binding SET domain protein 1 (*NSD1*) gene, an HMTase at 5q35.2–35.3. Here, we analyzed *NSD1* in 34 Brazilian Sotos patients and identified three novel and eight known mutations. Using protein modeling and bioinformatic approaches, we evaluated the effects of one novel (I2007F) and 21 previously reported missense mutations in the SET domain. For the I2007F mutation, we observed conformational change and loss of structural stability in Molecular Dynamics (MD) simulations which may lead to loss-of-function of the SET domain. For six mutations near the ligand-binding site we observed in simulations steric clashes with neighboring side chains near the substrate S-Adenosyl methionine (SAM) binding site, which may disrupt the enzymatic activity of NSD1. These results point to a structural mechanism underlying the pathology of the *NSD1* missense mutations in the SET domain in Sotos syndrome. *NSD1* mutations were identified in only 32% of the Brazilian Sotos patients in our study cohort suggesting other genes (including unknown disease genes) underlie the molecular etiology for the majority of these patients. Our studies also found NSD1 expression to be profound in human fetal brain and cerebellum, accounting for prenatal onset and hypoplasia of cerebellar vermis seen in Sotos syndrome.

## 1. Introduction

Sotos syndrome (MIM 117550), also known as cerebral gigantism with intellectual disability and delayed development of motor skills, was initially described in 1964, and hundreds of cases have been subsequently reported [1,2,3,4,5]. Most reported cases have been sporadic, but occasional familial cases have demonstrated that Sotos syndrome is also autosomal dominant [6,7]. Because of the phenotypic overlap, Sotos syndrome is sometimes difficult to distinguish from several other clinical disorders such as Weaver syndrome (MIM 277590), Bannayan–Riley–Ruvalcaba syndrome (MIM 153480) and Beckwith–Wiedemann syndrome (MIM 130650) [8]. Therefore, identification of a causative gene was necessary for the precise genetic diagnosis and understanding of the molecular and physiological basis of Sotos syndrome.

In 2002, positional cloning in a Sotos syndrome individual with a balanced translocation, t(5;8)(q35;q24.1), resulted in the identification of a nuclear receptor binding SET domain protein 1 (NSD1) located at the 5q35 breakpoint as a causative gene, and early studies have shown that haploinsufficiency of *NSD1* is the underlying cause of this syndrome [9,10]. To date, over 400 different mutations in *NSD1* associated with Sotos syndrome have been reported. Various mutations abrogating NSD1 function include missense mutations, partial gene deletions, microdeletions and truncating mutations resulting from small nucleotide insertions, deletions or splice-site mutations [3,4,11,12]. More than 80% of patients with Sotos syndrome carry *NSD1* mutations, whereas for 20% of phenotypically characterized cases with Sotos syndrome underlying aberrations in the *NSD1* gene have not been detected [5,13]. In particular, partial or whole *NSD1* gene 5q35 microdeletions are the most common cause of Sotos syndrome in Japanese patients. In non-Japanese patients, however, 5q35 microdeletions are uncommon, accounting for only 10% of affected individuals [4,11,14,15].

*NSD1* encodes a protein containing multiple functional domains including an SET [Su(var)3–9, Enhancer-of-zeste, Trithorax] domain, which is required for histone methyltransferase (HMTase) activity and thus present in most HMTases [16,17,18]. NSD1 also apparently contains five zinc finger-like plant homeodomain (PHD) domains and two proline-tryptophan-tryptophan-proline (PWWP) motifs, both of which are involved in protein–protein interactions [19,20]. However, the number of functional domains in NSD1, especially the PHD domain, has not been clearly defined yet [13,19,21,22]. NSD1 is a member of mammalian histone lysine methyltransferases that play important roles in multiple aspects of development and disease by acting as a transcriptional intermediary factor capable of both negatively and positively influencing transcription of nuclear receptors, such as the estrogen, retinoic acid and thyroid hormone receptors, depending on the cellular context [22,23,24,25].

Most *NSD1* missense mutations are present in the functional domains of NSD1, and to date a total of 21 missense mutations have been identified in the SET domain [4,5,11,13,26,27,28,29]. Although the SET domain has a crucial role in NSD1 function, the mechanisms by which these mutations result in loss-of-function have been poorly understood. In this study, we performed a systematic screening for *NSD1* mutations to evaluate the range and contribution of different *NSD1* aberrations to the spectrum of associated clinical features in Brazilian Sotos patients. In addition, using protein modeling and bioinformatic approaches, we investigated the impact of all reported missense mutations in the SET domain of the NSD1 protein. This study suggests that mutations in the SET domain of NSD1 may disrupt its HMTase activity by changing protein stability and interfering with its ability to bind a ligand.

## 2. Materials and Methods

### 2.1. Patients

A total of 34 Brazilian patients with Sotos syndrome were analyzed in this study. Each patient had been examined by a clinical geneticist or experienced pediatrician and satisfied strict criteria for a diagnosis of Sotos syndrome including facial features, macrosomia and neurological findings. We also checked the internal whole exome sequencing database of 600 healthy Brazilian controls to confirm that the mutations we found are not polymorphisms. For the screening of familial inheritance, parental and sibling DNA samples from seven families were also analyzed to see whether the novel mutations in four patients are de novo or inherited. Written informed consent forms were obtained from all involved patients. This study was approved by the Institutional Review Board of Augusta University and Hospital das Clínicas-São Paulo (CAPPesq number 0371/2010).

### 2.2. Genomic DNA Extraction

Genomic DNA was isolated from peripheral blood lymphocytes by standard methods of phenol/chloroform extraction and ethanol precipitation [30].

### 2.3. NSD1 Mutation Analysis

For each patient, exons 2 through 23 of the *NSD1* gene were amplified by polymerase chain reaction (PCR) using approximately 100 ng of genomic DNA and 2×AmpliTaq Gold^®^ PCR Mastermix (Life Technologies, Carlsbad, CA, USA) in a 25 µL reaction volume. PCR conditions were as follows: 94 °C for 4 min; 35 cycles of 94 °C for 30 s, 62 °C for 30 s, and 72 °C for 45 s; 72 °C for 5 min. Primer sequences are listed in Table S1 in Supplementary Materials. PCR products were purified using USB^®^ ExoSAP-IT^®^ reagent (Affymetrix, Santa Clara, CA, USA) following the manufacturer’s protocol, and sequenced using M13 primers and BigDye^®^ Terminator chemistry version 3.1 (Life Technologies) in a 10 µL reaction volume. DNA sequencing reaction conditions were as follows: 96 °C for 1 min; 25 cycles of 96 °C for 10 s, 50 °C for 5 s, and 60 °C for 1 min 15 s. Each product was then purified using either the Agencourt^®^ CleanSEQ^®^ dye terminator removal protocol on a Biomek^®^ FXP workstation (Beckman Coulter, Brea, CA, USA) or the EdgeBio Performa^®^ DTR V3 96-Well Short Plates method of dye terminator removal. Products were sequenced by the Sanger dideoxynucleotide method using an Applied Biosystems 3730XL DNA Analyzer instrument (Life Technologies). Sequence reads were aligned to reference sequence NM_022455.4 and analyzed for DNA sequence changes using Sequencher^®^ software version 4.9 DNA analysis software (Gene Codes Corporation, AnnArbor, MI, USA) and Sequence Pilot version 4.2.2 (JSI Medical Systems, Ettenheim, Germany) software. Any identified sequence alterations were queried against the Single Nucleotide Polymorphism database (dbSNP; NCBI; http://www.ncbi.nlm.nih.gov/SNP/) and the Human Gene Mutation Database (HGMD^®^ Professional; BIOBASE Biological Databases; https://portal.biobase-international.com).

### 2.4. Reverse Transcription-quantitative PCR (RT-qPCR)

Primers targeting *NSD1* exons 5 were designed for RT-qPCR (Table S2. Total RNA from whole human brain, fetal brain and from an additional 11 different central nervous system (CNS) regions (Clontech, Mountain View, CA, USA) were used to investigate *NSD1* transcripts levels. Complementary DNA (cDNA) was synthesized from 1 µg of total RNA using the RevertAid First cDNA Synthesis Kit (Thermo Scientific, Waltham, MA, USA). RT-qPCR assays were performed using 2 µL cDNA, 2.5 µM primer and 10 µL FastStart DNA Green Master (Roche, Indianapolis, IN, USA) in a 20 µL reaction volume.

### 2.5. Protein Modeling and Bioinformatics Analysis

Molecular Dynamics (MD) simulations were carried out using the program package GROMACS 4.5.4. [31]. The force field OPLS-AA [32] was used in all MD simulations. The crystal structure of the native SET domain (PDB ID: 3OOI) and the I2007F model (generated using MOE (Molecular Operating Environment, 2013.08; Chemical Computing Group Inc., Montreal, QC, Canada) without S-Adenosyl methionine (SAM) and histone peptide were used as a starting point for MD simulations. The native and mutant proteins were solvated with Simple Point Charge (SPC) water molecules [33]. Periodic boundary conditions were applied in an isothermal–isobaric (NPT) ensemble simulation. The system was neutralized by adding sodium ions and the temperature was controlled using a Berendsen thermostat [34] with a coupling time of 0.2 ps. The minimized system was equilibrated for 1000 ps each at 300 K in a position restrained molecular dynamics simulation in order to solvate the proteins. The equilibrated systems were then subjected to molecular dynamics simulations for 150 ns each at 300 K. From the trajectory we computed the distance between selected amino acids, which we plotted using the GRaphing, Advanced Computation and Exploration (GRACE) program (http://plasma-gate.weizmann.ac.il/Grace).

Some information regarding the effect of point mutations on the NSD1 protein function could already be obtained at the sequence level using in silico approaches. We used in silico tools including I-Mutant 2.0 [35], SPPIDER [36], Polyphen [37], PANTHER [38] and SIFT [39] (on all the mutations in Table 4) to calculate the relative residue stability constants for the wild-type (WT) protein and its mutant forms. These methods are trained on existing sets of mutations/phenotype association data and use sequence homologs, structural information, such as solvent accessible surface area and changes in amino acid properties, to provide information as input to machine learning methods for phenotype prediction.

## 3. Results

**Figure 1:**
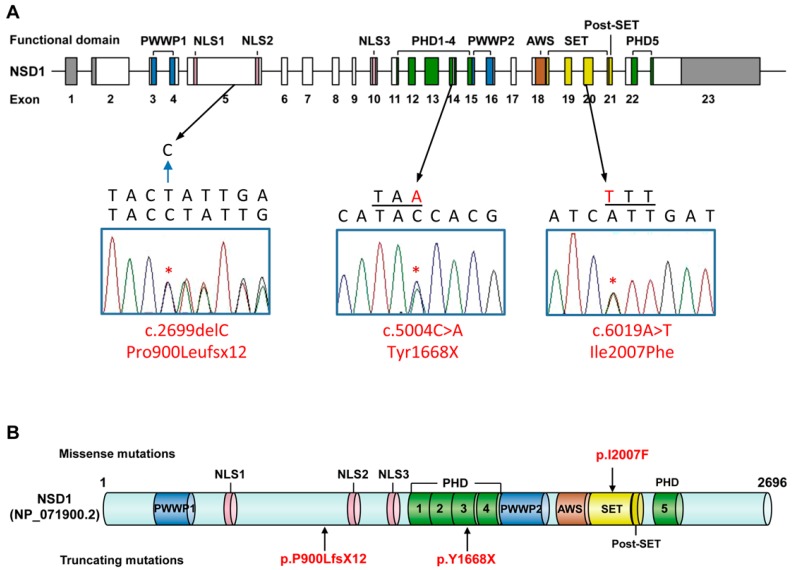
The location of three novel mutations in nuclear receptor binding SET domain protein 1 (*NSD1*). (**A**) Exonic structure of *NSD1* (NM_022455.4) with three novel mutations identified in Brazilian Sotos syndrome patients. Open and gray boxes indicate coding exons as well as the 5’ and 3’ untranslated regions, respectively. Specific functional domains are indicated by colored boxes, and chromatograms with nucleotide and amino acid sequences describe the novel mutations; (**B**) NSD1 functional domains and the localization of three novel mutations identified in our Brazilian Sotos syndrome patients. PWWP: proline-tryptophan-tryptophan-proline domain; NLS: nuclear localization signal; PHD: plant homeodomain domain; AWS: associated with SET domains; SET: Su(var)3–9, Enhancer-of-zeste, Trithorax domain. Positions of nuclear localization signals were from a previous report [21]. Figure is not to scale.

We first investigated the domain structure of NSD1 using bioinformatic approaches. The *NSD1* gene encodes a protein containing three PHD domains at amino acid positions 1543–1589, 1707–1751 and 2120–2160, predicted with high confidence using Pfam [40] and National Center for Biotechnology Information Basic Local Alignment Search tool (NCBI–BLAST) [41]. The SMART [42] server also predicts putative PHD domains at positions 1590–1639 and 1640–1693. However, a putative PHD domain at position 2164–2205 reported previously [19] was not found with this approach. The bioinformatics approach confirmed the existence of other domains, including one SET domain at amino acid position 1942–2065, two PWWP domains at positions 323–388 and 1756–1818, one associated with SET (AWS) domain at position 1890–1940 and one Post–SET domain at position 2066–2082, as previously reported (Figure 1A,B and Table 1) [19]. Three nuclear localization signals (NLSs) are located at amino acid positions 512–529, 1157–1174 and 1471–1488 [21].

**Figure 2:**
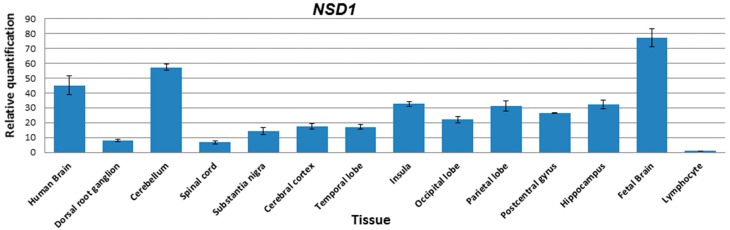
*NSD1* expression pattern in various regions of the brain. Transcripts of *NSD1* were detected in many regions of the brain including the hippocampus. The highest transcript levels were detected in fetal brain (~75-fold), while the cerebellum also showed high levels of transcript (~60-fold) relative to lymphocytes. *NSD1* transcripts in the dorsal root ganglion and spinal cord were much lower compared to other regions assayed.

Assaying the expression levels of *NSD1* mRNA in various regions of the brain by RT-qPCR revealed that the gene is highly expressed throughout the brain. Approximately 60-fold higher expression was detected in the cerebellum, while ~30-fold higher transcript levels were recorded in the insula, parietal lobe and hippocampus compared to lymphocytes. Fetal brain showed ~75-fold higher transcript levels relative to lymphocytes. The lowest levels of *NSD1* transcripts were detected in the dorsal root ganglion and spinal cord (Figure 2).

Genomic DNAs samples from 34 Brazilian patients with phenotypes of Sotos or Sotos-like syndrome as well as DNA samples from unaffected members of seven unrelated families were screened for intragenic *NSD1* mutations by Sanger sequencing analysis. We identified *NSD1* mutations in 11 out of 34 patients, including three novel mutations (Table 2). In our patient DGDP186, one novel nonsense mutation (c.5004C>A; NM_022455.4) was identified in exon 14 resulting in the substitution of a tyrosine by a premature stop codon TAA at amino acid position 1668 in the third PHD domain (p.Y1668X; NP_071900.2, Figure 1A, Table 2). This substitution results in the premature truncation of the NSD1 protein with the deletion of C-terminal functional domains of NSD1 including the PWWP2, AWS, SET and PHD domains 3–5 (Figure 1B). Parental DNA analysis demonstrated that the c.5004C>A (p.Y1668X) mutation in this patient is de novo.

**Figure 3:**
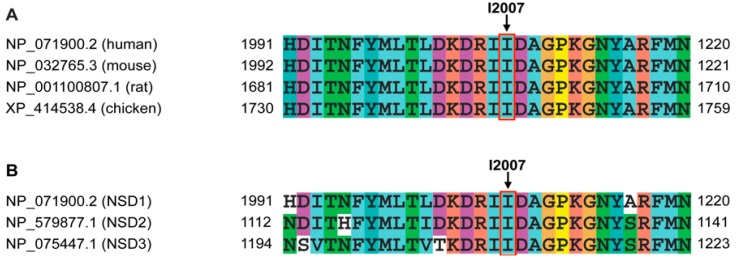
Multiple amino acid sequence alignment of NSD1 vertebrate orthologs and paralogs, in which I2007 shows a full invariant conservation. (**A**) Alignment of NSD1 orthologs from the *Gallus gallus*, *Mus musculus* and *Rattus norvegicus*. Amino acid residues surrounding p.I2007 in vertebrate orthologs are evolutionarily fully conserved, suggesting the functional importance of the SET domain; (**B**) Alignment of human NSD1, NSD2 and NSD3. Amino acid sequence alignment of NSD1 paralogs show partial consensus, indicating their differential functions.

One missense mutation (c.6019A>T, p.I2007F) was identified in patient DGDP291 (Figure 1A, Table 2). This alteration occurs within the highly conserved SET domain (Figure 1B), where it may have critical effects on the histone methyltransferase activity of NSD1. Paternal DNA was unavailable for analysis; however, the familial genetic analysis including the healthy mother and unaffected brother revealed that this mutation was likely de novo, and this mutation was not present in the other 34 patients or the more than 600 Brazilian control individuals. This change is also not present in the Exome Aggregation Consortium (ExAC) database [43]. Analysis of its position within *NSD1* provides further evidence supporting a pathogenic role for this missense mutation. This alteration occurs at amino acid residue 2007 within the functional domain that is conserved not only in other species such as mice, rat and chicken, but also in the known human paralogs of *NSD1*, *NSD2* and *NSD3* (Figure 3).

In another patient, DGDP306, a one base-pair deletion in exon 5 of the *NSD1* gene (c.2699delC) was identified which leads to a frameshift mutation resulting in the premature termination of protein translation at amino acid 911 (p.P900LfsX12; Figure 1A, Table 2). The remaining protein fragment contains only the N-terminal region of the NSD1 protein, including one functional PWWP domain (Figure 1B). This may lead to loss-of-function of the protein causing Soto syndrome. Analysis of parental samples indicated that the mutation had occurred de novo. Since two of the novel mutations were predicted to result in premature truncation of the NSD1 protein because of either nonsense or frameshift mutations, these two mutations are considered to be pathogenic.

Additionally, eight previously-reported mutations including three frameshift mutations (p.S985CfsX25 [13], p.K1002EfsX8 [44] and p.K1580NfsX62 [45]), two nonsense mutations (p.R632X [1] and p.Q1989X [4]), one splice-site mutation (c.5892+1G>T, exon 19 skipping [5]) and two missense mutations (p.L1917P [46] and p.R2017Q [11]) were identified in our Sotos patients. Among them, two mutations, c.4740delA and c.5892+1G>T, were de novo in patients DGDP173 and DGDP176, respectively, whose parental samples were available. The nomenclature of the previously reported frameshift mutation p.K1002GfsX8 [44] has been corrected herein to p.K1002EfsX8. We also observed 16 polymorphisms (Table S3). These were considered nonpathogenic, since they were already identified in multiple individuals including those in healthy populations. Since exons 5 (2560 bp) and 23 (1628 bp without 3’-untranslated region (UTR)) are very large, a number of polymorphisms have been identified in these two exons. The polymorphisms we identified in our patients were also located only in exons 5 and 23, except for one single nucleotide polymorphism (SNP) rs79098301 in intron 17 (Table S2). Interestingly, one synonymous polymorphism rs28580074 in exon 23 (c.6829T>C, p.L2277L) was observed in all of our 34 Sotos patients, of which 26 patients were homozygous for this change.

**Figure 4:**
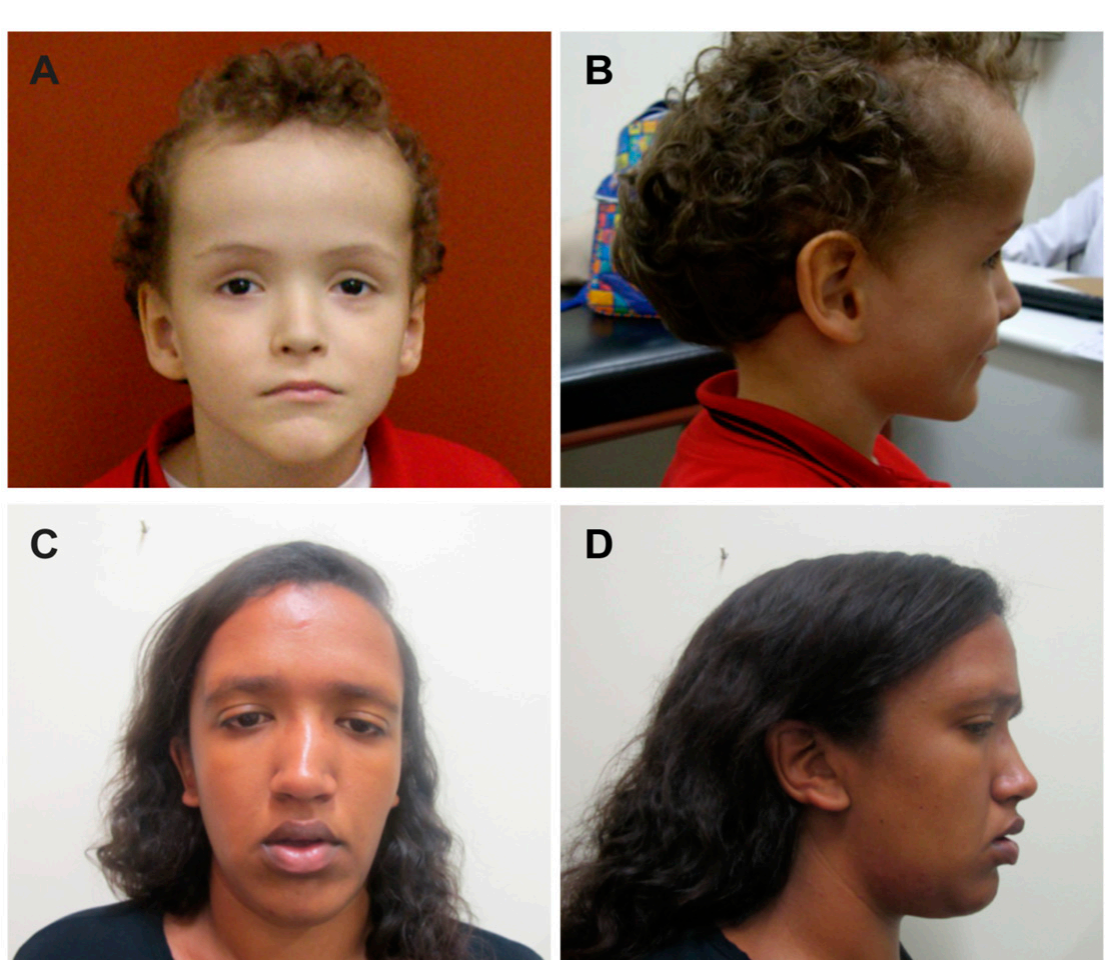
General features in Brazilian Sotos syndrome patients with *NSD1* mutations. (**A**,**B**) Facial features of DGDP186 at age eight years with macrocephaly, prominent forehead, ocular hypertelorism, downslanting palpebral fissures and pointed chin; and (**C**,**D**) facial features of DGDP291 at age 21 years with macrocephaly, prominent forehead, ocular hypertelorism, downslanting palpebral fissures and pointed chin.

The clinical features of the patients with novel mutations are summarized in Table 2. Our patient DGDP186 with a novel nonsense mutation has a typical facial gestalt in association with a variable number of additional major criteria including overgrowth, developmental delay and facial dysmorphism (Figure 4A,B). Although this patient did not show advanced bone age, one of the major diagnostic criteria for Sotos syndrome, his other phenotypic features satisfy other major diagnostic criteria such as macrocephaly, post-natal overgrowth and delayed milestones. Another patient DGDP306 with a novel frameshift mutation also has the typical facial dysmorphisms observed in other Sotos patients. The neurologic involvement seems to be predominant in this patient characterized by hypotonia, seizures and increased subarachnoid spaces. This girl had a markedly advanced bone age (her bone age was three years six months at one year eight months of age). This patient shows excessive growth velocity compared to other control populations at the same age. In addition, her developmental milestones are delayed. The third Sotos patient DGDP291 with a novel *NSD1* mutation also shows a typical Sotos phenotype including post-natal overgrowth, developmental delay and facial gestalt (Figure 4C,D). She has advanced bone age and macrocephaly as well as delayed learning ability. The phenotypic features of other Sotos patients with reported *NSD1* mutations are also summarized in Table 3.

**Figure 5:**
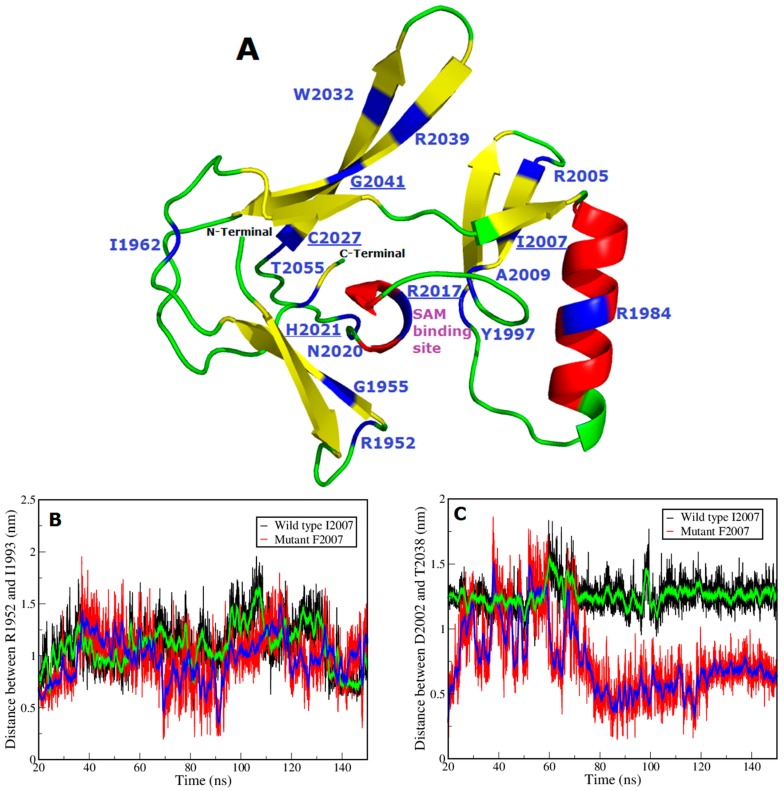
Inhibitory effect of the novel mutation, p.I2007F on ligand-binding properties. (**A**) Structural model for the human NSD1 SET domain protein (1946–2063 amino acids) in complex with S-Adenosyl methionine (SAM) (PDB ID: 3OOI). Color code: red indicates helix; yellow specifies strands; green signifies loops; and blue represents positions of the missense mutation sites including our novel mutation p.I2007F in Table 4. Residues p.R1952, p.Y1997, p.R2017, p.N2020, and p.H2021 interact with SAM, which is bound in a pocket formed by the SET domain. The SAM binding site is also labeled. Six mutations (p.I2007F, p.R2017Q, p.R2017W, p.H2021R, p.C2027R and p.G2041D) which might cause steric clashes with neighboring amino acid side chains are also shown in blue and underlined; (**B,C**) Molecular Dynamics (MD) Trajectory analysis of the wild-type (WT) (in black) and p.I2007F mutant (in red); where X-axis is the length of the simulation in nanoseconds (ns) and Y-axis is the distance in nano-meter (nm); (**B**) Distance D1 between residues p.R1952 and p.I1993 in the ligand-binding site for native type (black line) and mutant model (red line). Substitution of phenylalanine shrinks the distance D1 from a range of 10.3–16.9 Å (D1 = 10.48 ± 1.88 Å) to a range of 3.4–13.7 Å (D1 = 9.79 ± 1.97 Å); (**C**) Distance D2 between residues p.D2002 and p.T2038 in the ligand-binding site for native type (black line) and mutant model (red line). Substitution of phenylalanine shrinks the distance D2 from a range of 10.8–15.3 Å (D2 = 12.60 ± 0.82 Å) to a range of 3.0–7.0 Å (D2 = 6.67 ± 1.22 Å). Therefore, the mutation from isoleucine to phenylalanine at amino acid position 2007 significantly reduces the size of the binding pocket that strongly affects ligand-binding properties.

**Figure 6:**
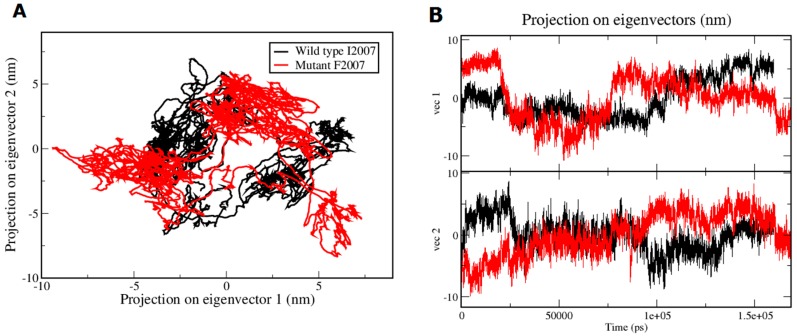
PCA scatter plot along first two principal components, PC1 and PC2 of WT and I2007F mutant. (**A**) Show the differences in the motion between wild type (in black) and I2007F mutant (in red) over the simulation time; (**B**) Projection of the eigenvectors (vectors 1 and 2) along Y-axis and time on X-axis (in picoseconds; ps).

To explore the effect of I2007F point mutation in the SET domain of the protein encoded by *NSD1*, we generated a mutant structural model using sequence alignment (ClustalW) [47] based on the homology to the human histone-lysine *N*-methyltransferase NSD1 SET domain (PDB ID: 3OOI) [48] (116 amino acids; spanning the amino acid sequence 1942–2063) as template protein. Figure 5A shows the model for the SET domain which is composed of three β-sheets (yellow) arranged in a triangular fashion and one long helix (red) exposed to the outside. The novel mutation p.I2007F is predicted to be non-tolerable using SIFT server [39,49,50,51,52] and is predicted to decrease the stability of the protein with a negative DDG (delta delta G/free energy change) value of −1.65. This mutation is located in the evolutionarily fully conserved SET domain, with both WT and mutant residues interacting with surrounding amino acids. Mutation of this invariant residue to phenylalanine likely causes steric clashes with neighboring side chains, as phenylalanine is bulkier than isoleucine, and can affect the SAM ligand-binding pocket. To verify this assumption, we performed MD simulations on the wild type SET domain of the NSD1 protein and the protein with the p.I2007F alteration to predict the conformational change induced by the mutation in and around the ligand binding site. Figure 5B (black line) illustrates the distance D1 between native p.R1952 and p.I1993 residues in the ligand binding pocket and Figure 5C (black line) illustrates the distance D2 between native p.D2002 and p.T2038 residues around the ligand-binding pocket. The WT protein maintains a distance in the range of 10.3–16.9 Å for D1 (D1 (average) = 10.48 ± 1.88 Å) and distance in the range of 10.8–15.3 Å (D2 (average) = 12.60 ± 0.82 Å) for D2. We also measured the distances between these positions in the crystal structure of the human histone-lysine *N*-methyltransferase NSD1 SET domain (PDB ID: 3OOI) which was D1 = 13.8 Å and D2 = 8.2 Å. In the p.I2007F mutant the distance D1 shrinks to a range of 3.4–13.7 Å (D1 (average) = 9.79 ± 1.97 Å) and the D2 to a range of 3–7 Å (D2 (average) = 6.67 ± 1.22 Å). Substitution of isoleucine to phenylalanine reduces the size of the binding pocket that can strongly affect the ligand/histone peptide-binding properties. We also performed principal component analysis (PCA) on the trajectories. The PCA scatter plot for the WT and I2007F mutant in Figure 6A,B shows a significant difference in the motion of both systems as evident from the characteristic structures plotted along the direction of two principal components. From the PCA scatter plot it is clear that the eigenvectors computed from the MD trajectories for both the systems, the WT and p.I2007F mutant (Figure 6), differ significantly. This indicates the difference in protein motion between the WT and the mutant protein. The most pronounced flexibility observed in the WT protein was around residues I2007–M2020 (loop), which flips the residues K2013 and Y2016 towards the SAM binding pocket. The residues R2018 and M2020 in this loop region are known to interact with K20 of H4 peptide through hydrogen bonding [53] thus stabilizing the complex. A similar conserved hydrogen bonding is observed amongst all lysine-HMTases (H4K20-SETD8, H3K4-SETD7, H3K27-vSET, GLP and Dim-5). However, this motion is not observed in the pI2007F mutant, the significant structural motion observed in the mutant occurs in the vicinity of the K2031-G2041 loop. This is also supported by the difference in the distance between residues D2003–T2038 in the Figure 5C. From the analysis on WT and I2007F mutant, it is clear that the p.I2007F mutation causes changes in the conformational state of the protein and the motion observed during the simulation, suggest change in and around the binding pocket in the mutant state (closed state) as compared to the open state in WT protein. It is likely that the loop flipping around residues I2007–R2017 (loop) might participate in the H4 peptide binding to the SET domain. However, in our simulations, the SAM co-factor and H4 peptide are not included in the complex, so these interactions are not possible to observe.

A number of point mutations have been previously reported in the SET domain, including p.R1952W, p.G1955D, p.Y1997S, p.C2027R, p.G2041D, p.Y2058C [4], p.I1962T, p.R1984G [26], p.R1984Q, p.Y1997C, p.R2017W [27], p.Y1997H, p.N2020S [13], p.R2005Q, p.R2017Q [11], p.A2009V, p.H2021R, p.C2027Y, p.W2032L [5], p.R2039C [29] and p.T2055I [28]. Figure 5A shows the positions of the point mutations in the SET domain model. We performed a bioinformatics-based analysis using SPPIDER [36], PolyPhen2 [37] and SIFT [49] on the effect of all of these mutations (Table 4). We also used the HMM based evolutionary approach PANTHER to investigate the impact of point mutations on protein function [38]. Using the HMM based evolutionary approach, 18 out of 22 point mutations were designated as deleterious with subPSEC (substitution position-specific evolutionary conservation) scores in the range of −9.24 to −3.04. Four other mutations exhibited a subPSEC score in the range of −1.96 to −2.97. The structural model of the human SET domain (PDB ID: 3OOI) allowed us to observe the interactions of residues p.R1952, p.Y1997, p.R2017, p.N2020, p.N2065 and p.H2021 with the SAM [48], which is bound in a pocket formed by the SET domain (Figure 5A). SAM is a common co-substrate involved in methyl group transfers in a number of biological processes, and changes in the structural stability due to mutations near the ligand-binding site could affect the interaction of the NSD1 protein with the ligand SAM [48]. In order to investigate this possibility, we generated homology models of the SET domain with the 22 missense mutations listed in Table 4 (shown in Figure 5A, labeled as blue). Mutations p.I2007F, p.R2017W, p.R2017Q, p.H2021R, p.C2027R and p.G2041D may cause steric clashes with neighboring amino acid side chains or may affect the water-mediated hydrogen bond [54,55] with SAM, thus likely affecting the structural stability of the protein. The activity of four *NSD1* mutations p.R1952W, p.R1984Q, p.R2017W and p.Y1997C in the SET domain was investigated [56]. For all four mutations, the results showed complete loss of the protein’s ability to methylate histone for all the four mutations, which is in accordance with the findings published for p.R1952W, p.R1984Q, and p.R2017Q [48]. Using SIFT analysis we also analyzed whether these missense mutations in the SET domain may affect the protein function based on sequence homology and the physical properties of amino acids. This tool calculates a score for every substitution and predicts the functional effect. All SET domain mutations were predicted to be non-tolerable and to affect protein function with scores < 0.05 (Table 4).

We further analyzed these mutations using PolyPhen2 [37], which uses information about the structure of the protein (hydrophobicity, charge effects and charges in molecular contacts) and multiple sequence alignment to predict the effect of mutations based on the Position Specific Independent Count Score difference. Based on the calculated alignment score and difference in structural parameters, PolyPhen2 designated all 22 missense variants as being “probably damaging” with a score of 1 or very close to it. In order to improve prediction accuracy of structure based tools, we also used the support vector machine based tool I-Mutant 2.0 [35], which predicts the effect of point mutations on protein stability. Seventeen missense mutations including p.I2007F are predicted to decrease stability with negative scores among 22 variants (Table 4). In addition we used SPPIDER [36] analysis to determine whether sites affected by these mutations are possible binding sites in the NSD1 sequence, as such sites are likely to affect protein function and signaling. Although SPPIDER did not predict any residue to be an interaction site (Table 4), I-Mutant 2.0 suggested that these mutations might increase or decrease the stability of the protein, indicating the deleterious effects of these mutations.

## 4. Discussion

Because of the phenotypic overlap with other groups of overgrowth syndromes, such as Weaver, Bannayan–Riley–Ruvalcaba and Beckwith–Wiedemann syndromes, Sotos syndrome may be challenging to diagnose [8]. Although the presence of the three major criteria and several other conditions associated with Sotos syndrome allow us to infer a clinical diagnosis of typical Sotos syndrome, the genetic analysis of the *NSD1* gene is considered another criterion for the confirmation of the molecular diagnosis. *NSD1* mutations are found in 70%–93% of classical cases of Sotos syndrome, but the molecular basis is unknown for remaining patients [11,14,15,57]. In our study, we have identified 11 different *NSD1* mutations including three novel ones in 11 Brazilian Sotos patients. The prevalence of *NSD1* mutations in our Brazilian Sotos patients was lower (32%, 11 out of 34 patients) compared to the frequency reported for other populations. Although the whole genome microarray did not identify any large 5q35 microdeletions involving the *NSD1* gene in the 23 patients that were negative by *NSD1* sequencing analysis, additional studies such as Multiplex Ligation-dependent Probe Amplification (MLPA) or targeted high density SNP array need to be performed to rule out the presence of smaller *NSD1* deletions. Therefore, this low prevalence of *NSD1* intragenic mutations in Brazilian Sotos patients is probably due to ethnic differences, a different frequency of the microdeletion, mutations in non-coding regions or mutations in other genes.

The mutations abrogating *NSD1* function include missense mutations, nonsense mutations, small intragenic insertions/deletions, splicing defects and microdeletions, which are distributed over the entire gene including both coding and non-coding sequences [1,4,11]. Chromosomal microdeletions involving the *NSD1* gene have been described as a major cause of Sotos syndrome in Japanese patients (prevalence >45%) [14], but are less frequently found in non-Japanese patients (approximately 10% of prevalence), where intragenic point mutations are highly prevalent [4,11,57]. The reason for this discrepancy is not known, but the presence of low-copy repeats (LCRs), highly homologous sequences, in regions flanking proximal and distal breakpoints of the common deletion suggests that LCRs may mediate the deletion [14]. A previous study identified one patient with total deletion of *NSD1* and two patients with partial deletion of *NSD1* among 30 Brazilian patients with the clinical diagnosis of Sotos syndrome [58], suggesting that the prevalence of microdeletions involving *NSD1* is likely to be low in Brazilian Sotos patients. Among the 11 patients with 11 different *NSD1* mutations in our study, three patients had *NSD1* mutations, including one missense, one nonsense and one-frameshift mutation.

In agreement with previous studies in other populations, the phenotypic characterization of Brazilian Sotos patients with a mutant *NSD1* genotype suggests that the most consistent diagnostic criterion in Sotos syndrome is represented by facial dysmorphism, in association with a variable number of additional major criteria [1,11,12,59]. As summarized in Table 2 and Table 3, most *NSD1* mutant Sotos patients exhibited a typical facial gestalt including macrocephaly (10/11, 91%), prominent forehead (11/11, 100%), ocular hypertelorism (11/11, 100%) and pointed chin (11/11, 100%). Other frequent features among our patients with intragenic mutations were the presence of hypotonia (11/11, 100%), excessive growth velocity (10/11, 91%) and delayed learning ability (8/9, 89%). Advanced bone age, one of the major diagnostic criteria for Sotos syndrome, was not significant in five patients. In contrast, other criteria including seizures, heart defects and intellectual disability were inconsistent features, although most patients display delayed developmental milestones. We also tried to compare phenotypes in our Sotos patients to other reported cases with the same mutations. Due to the unavailability of the detailed phenotypic information of the reported cases with the same mutations identified by our study, however, it was not possible to establish a correlation between clinical features and the type of mutations. More data should be accumulated to explain appropriate genotype-phenotype correlations and ethnic difference in the prevalence of different *NSD1* mutations.

NSD1 contains multiple functional domains including a zinc finger-like PHD domain, a PWWP motif and a SET domain. In an earlier study, NSD1 had been reported to contain six functional domains (PHD-1, PHD-2, PHD-3, PWWP-1, PWWP-2 and SET) [22]. However, a recent study proposed the existence of additional PHD domains (two canonical and one putative) in NSD1 [19]. Therefore, the number of functional domains in NSD1, especially the PHD domains, has not been clearly defined yet. Our bioinformatics analyses suggested that NSD1 contains five PHD domains (Table 1, Figure 1B). However, a putative PHD domain (amino acid positions 2164–2205) reported previously [19] could not be identified. Other functional domains including two PWWP motifs, one SET domain and one post-SET domain also appeared present in NSD1, and their locations highly overlap with previous reports [19,22].

NSD1 is a family member of histone methyltrasferases and regulates the expression of target genes through the post-translational modification of histones. Thus, disruption of HMTase activity in NSD1 could have severe phenotypic consequences [60]. Studies have shown that NSD1 methylates on histone H3 lysine 36 (H3K36), H4 lysine 44 (H4K44) and H1.5 lysine 168 (H1.5K168) [24,61]. NSD1 has also been shown to activate nuclear factor-κB (NF-κB)-p65, a subunit of the NF-κB transcription complex through K218/K221 methylation. NF-κB is a central coordinator of immune and inflammatory responses [62]. Furthermore, translocations involving *NSD1* have been found in patients with acute myeloid leukemia (AML) and breast cancer, indicating that NSD1 may function in the regulation of multiple downstream genes involved in various processes, such as bone morphogenesis, development and cancer [61,63]. A recent in vitro study showed that PHD domains in NSD1 bind mono-, di- or tri-methylated histones, H3K4 and H3K9, and point mutations in PHD domains would disrupt the transcriptional regulation of NSD1 by compromising the binding to these methylated lysines and to the transcription cofactor Nizp1 [19].

Twenty-one missense mutations have been previously reported in the SET domain [5,11,13,26,27,28,29,50] and one of the novel missense mutations we identified, c.6019A>T (p.I2007F), also occurred at a highly conserved amino acid in the SET domain (Figure 3). The SET domain is required for methyltransferase activity in all HMTases that are critical for genetic regulation at the epigenetic level [64,65]. Several different mechanisms have been proposed, but there has been no consensus regarding the understanding of the catalytic mechanism upon the transfer of the methyl group by the SET domain. However, the recently determined three-dimensional structures of the SET domains from chromosomal proteins support the notion that SAM and the lysine residue of the substrate histone tail must first be bound with a proper orientation in the catalytic pocket of the SET domain. Then, methyl transfer from SAM to the ε-amino of the target lysine likely proceeds by a direct in-line S_N_2 nucleophilic attack through a nearby tyrosine residue [66,67,68]. Several different bioinformatics analyses suggested that all 22 missense mutations in the SET domain would have deleterious effects, which may be induced by changes in the functional activity of this domain, by changing protein stability or by interfering with the ability to bind to a ligand, among other factors. These data are in agreement with published results [48,56]. Specifically, we have investigated p.I2007F, where the side chain of phenylalanine is significantly larger than that of isoleucine, pushing neighboring side chains outwards, possibly into the binding pocket. As a result, the ligand may either completely or partially lose its affinity with the protein, which would correspond to a significant loss-of-function of this domain. This computational result has yet to be confirmed by experimental data. However, SIFT analysis suggested that this mutation would affect the protein function, supported by molecular dynamics simulation results which revealed that it would significantly reduce the size of the binding pocket that strongly affects ligand-binding properties. This result is consistent with the previous observation that the Sotos mutation involving five arginine residues in the SET domain had greatly reduced H3K36 methyltransferase activities in vitro; some of them showed almost undetectable enzymatic activities [48]. Therefore, it may have significant deleterious effects on the methyltransferase activity of NSD1 resulting in altered gene expression patterns by the deregulation of histone modifications at H3K36 and H4K44 [48]. Moreover, uncontrolled gene expression resulting from mutations in genes encoding histone modifiers including histone acetyltransferases, deacetylases, methyltransferases and kinases causes developmental defects, in particular those involving the heart and nervous system, as well as cancers [69,70,71]. In fact, some types of human congenital diseases have been shown to involve the abnormal expression or activity of a family of histone methyltransferases. These include NSD2/ Wolf-Hirschhorn Syndrome Candidate 1 (WHSC1) in Wolf-Hirschhorn syndrome, NSD3 in cancer, euchromatin histone methyl transferase1 (EHMT1) in the 9q subtelomeric deletion syndrome and enhancer of zeste, drosophilahomolog 2 (EZH2) in Weaver syndrome [72,73,74,75]. More recently, mutations *DNMT3A*, another DNA methyltransferase involved in the de novo methylation of DNA were also identified in patients with overgrowth syndromes [76]. Our data combined with two previous published data sets [48] suggest that point mutations in the NSD1 SET domain disrupt its transcriptional activity by interfering with its ability to bind a ligand required for methyl group transfer and by changing protein stability. Therefore, highly conserved amino acids in the SET domain are critical for the transcriptional activity of NSD1 required for the regulation of gene expression of a number of downstream targets. Abrogation of this NSD1 transcriptional regulation would result in the multiple phenotypes observed in Sotos patients.

NSD1 was reported to be expressed strongly in fetal brain, kidney, skeletal muscle, spleen and the thymus in Northern blot analysis [22]. However, little is known about its expression in various brain regions. Using RT-qPCR, we examined the *NSD1* expression pattern in human brain, fetal brain, and additional 11 CNS human tissues compared to lymphocyte (Figure 2). Among the tissues examined, fetal brain displayed the highest *NSD1* expression, suggesting a fetal onset of the neurological phenotype of this congenital disorder. It also showed high expression in cerebellum, which is commensurate with the hypoplasia of cerebellar vermis in Sotos syndrome [13].

Since *NSD1* mutations have not been detected in 7%–30% of Sotos patients, other genes were also screened in some cases of unexplained Sotos syndrome. Because of their high sequence similarities with *NSD1*, both the *NSD2* and *NSD3* genes were tested previously as potential candidates for *NSD1*-negative Sotos syndrome cases [77]. However, no deletions were identified in 78 overgrowth syndrome cases without *NSD1*-mutation. Two missense alterations in *NSD2* were identified in two non-Sotos overgrowth cases but neither was within a functional domain [77]. This group also identified three synonymous and two intronic variants in *NSD2* and two synonymous substitutions in *NSD3*. Intragenic deletion of glypican 3 (*GPC3*) involved in Simpson–Golabi–Behmel syndrome was identified in a patient who was originally diagnosed with Sotos syndrome [78,79], and two 11p15 anomalies including paternal uniparental disomy and epigenetic defects (abnormal methylation status) were also identified among 20 patients with Sotos syndrome [80]. However, the molecular defect underlying a significant proportion of sporadic Sotos cases still remains unknown. In particular, in our Brazilian Sotos cohort, 68% of Sotos syndrome patients represented in 23 families have an unknown etiology. Therefore, these *NSD1* mutation-negative families will surely contribute to the identification of new genes involved in Sotos syndrome in the future.

## Figures and Tables

**Table 1:** Locations of functional domains in NSD1.

Domains	Amino acid Positions
PWWP1	323–388
NLS1	512–529
NLS2	1157–1174
NLS3	1471–1488
PHD1	1543–1589
PHD2	1590–1639
PHD3	1640–1693
PHD4	1707–1751
PWWP2	1756–1818
AWS	1890–1940
SET	1942–2065
Post-SET	2066–2082
PHD5	2120–2160

PWWP: proline-tryptophan-tryptophan-proline domain; NLS: nuclear localization signal; PHD: plant homeodomain domain; AWS: associated with SET domains; SET: Su(var)3–9, Enhancer-of-zeste, Trithorax domain.

**Table 2:** Clinical features in Brazilian Sotos patients with novel *NSD1* mutations.

Patient	DGDP186 (Male)	DGDP 291 (Female)	DGDP306 (Female)
Age at diagnosis	9 months	2 years 7 months	1 year 5 months
Current age	10 years	23 year	5 years
Nucleotide change (NM_022455.4)	c.5004C>A	c.6019A>T	c.2699delC
Exon	14	20	5
Amino acid change (NP_071900.2)	p.Y1668X	p.I2007F	p.P900LfsX12
Familial analysis	de novo	Both mother and brother with no variant, father unavailable	de novo
Brazilian control analysis	not found in 600 controls	not found in 600 controls	not found in 600 controls
**Developmental Milestone**			
Prenatal overgrowth	−	−	−
Excessive growth velocity	+	+	+
Advanced bone age	−	+	+
Large hands and feet	+	+	+
Developmental delay	Mild	Mild	Mild
Lack of fine motor control	−	−	−
Walking at	22 months	24 months	23 months
Speaking	Delayed	Delayed	Delayed
Delayed learning ability	−	+	N/A
**Facial Dysmorphism**			
Macrocephaly	+	+	+
Prominent forehead	+	+	+
High-arched palate	+	−	+
Pointed chin	+	+	+
**Other Features**			
Hypotonia	+	+	+
Seizures	−	−	+
Scoliosis	+	+	−
Enlarged ventricles	+	N/A	+
Artrial septal defect	−	+	−
Pulmonary stenosis	−	+	−

N/A, not applicable.

**Table 3:** Clinical features in Brazilian Sotos patients with known *NSD1* mutations.

Patient	DGDP168	DGDP171	DGDP173	DGDP174	DGDP176	DGDP180	DGDP183	DGDP305
Age at diagnosis	7 years	14 years 3 months	8 years 3 months	9 months	1 years 6 months	1 years 9 months	7 years	2 years 8 months
Current age	25 years	27 years	18 years	13 years	20 years	15 years	18 years	7 years
Nucleotide change (NM_022455.4)	3004_3005 delAA	5750T>A	4740delA	6050G>A	5892+1G>T	2954_2955 delCT	1894C>T	5965C>T
Exon or intron	5	18	12	20	Intron 19	5	5	19
Amino acid change (NP_071900.2)	p.K1002EfsX8	p.L1917P	p.K1580NfsX62	p.R2017Q	Exon 19 skipping	p.S985CfsX25	p.R632X	p.Q1989X
**Developmental Milestone**								
Prenatal overgrowth	+	+	+	−	+	−	−	+
Excessive growth velocity	+	+	+	−	+	+	+	+
Advanced bone age	−	+	+	+	+	+	−	+
Large hands and feet	+	+	+	+	+	+	+	+
Developmental delay	Mild	Mild	Mild	Mild	Mild	Mild	Mild	Mild
Lack of fine motor control	−	+	+	+	+	+	−	+
Walking at	15 months	24 months	18 months	20 months	17 months	20 months	Delayed	20 months
Speaking	Delayed	Delayed	Normal	Delayed	Delayed	Delayed	Delayed	Normal
Delayed learning ability	+	+	+	+	+	+	+	+
**Facial Dysmorphism**								
Macrocephaly	+	+	+	−	+	+	+	+
Prominent forehead	+	+	+	+	+	+	+	+
High-arched palate	−	+	+	+	+	+	+	+
Pointed chin	+	+	+	+	+	+	+	+
**Other features**								
Hypotonia	+	+	+	+	+	+	+	+
Seizures	−	−	−	−	−	−	+	−
Scoliosis	−	−	−	−	+	+	−	+
Enlarged ventricles	+	+	+	+	+	−	−	−

**Table 4:** Summary of the predictions of the bioinformatics tools PolyPhen 2, SPPIDER, SIFT, I-MUTANT 2.0 and PANTHER, with regard to the effect of point mutations in the *NSD1* gene.

AA Position	Polyphen2 (Predicted Impact)	PolyPhen2 (PSIC Score)	SPPIDER	SIFT Predicted Impact (Tolerance Index)	DDG Value Kcal/Mol	I-Mutant 2.0 Predicted Impact	PANTHER (subPSEC)
p.R1952W	Probably damaging	0.999	Non-interacting	Intolerant (0.00)	0.18	Increase Stability	−3.04478
p.G1955D	Probably damaging	1.000	Non-interacting	Intolerant (0.00)	−1.47	Decrease Stability	−7.27103
p.I1962T	Possibly damaging	0.934	Non-interacting	Intolerant (0.00)	−0.95	Decrease Stability	−2.82147
p.R1984G	Probably damaging	1.000	Non-interacting	Intolerant (0.00)	−0.93	Decrease Stability	−6.53802
p.R1984Q	Probably damaging	1.000	Non-interacting	Intolerant (0.00)	−0.53	Decrease Stability	−6.59809
p.Y1997H	Probably damaging	1.000	Non-interacting	Intolerant (0.00)	−0.95	Decrease Stability	−3.31488
p.Y1997S	Probably damaging	0.999	Non-interacting	Intolerant (0.00)	−0.70	Decrease Stability	−3.43065
p.Y1997C	Probably damaging	1.000	Non-interacting	Intolerant (0.00)	−1.22	Decrease Stability	−4.51444
p.R2005Q	Probably damaging	1.000	Non-interacting	Intolerant (0.01)	−1.13	Decrease Stability	−1.95697
p.I2007F	Probably damaging	1.000	Non-interacting	Intolerant (0.00)	−1.65	Decrease Stability	−2.97211
p.A2009V	Probably damaging	1.000	Non-interacting	Intolerant (0.00)	−1.25	Decrease Stability	−2.74529
p.R2017Q	Probably damaging	1.000	Non-interacting	Intolerant (0.00)	−0.27	Decrease Stability	−6.4321
p.R2017W	Probably damaging	1.000	Non-interacting	Intolerant (0.00)	0.02	Increase Stability	−8.09732
p.N2020S	Probably damaging	1.000	Non-interacting	Intolerant (0.00)	−0.81	Decrease Stability	−6.32023
p.H2021R	Probably damaging	1.000	Non-interacting	Intolerant (0.01)	0.16	Increase Stability	−9.24832
p.C2027R	Probably damaging	1.000	Non-interacting	Intolerant (0.00)	0.31	Increase Stability	−3.94723
p.C2027Y	Probably damaging	1.000	Non-interacting	Intolerant (0.00)	−0.52	Decrease Stability	−4.23261
p.W2032L	Probably damaging	0.999	Non-interacting	Intolerant (0.001)	−1.03	Decrease Stability	−6.15612
p.R2039C	Probably damaging	1.000	Non-interacting	Intolerant (0.00)	−1.58	Decrease Stability	−3.90311
p.G2041D	Probably damaging	1.000	Non-interacting	Intolerant (0.00)	−2.27	Decrease Stability	−3.7109
p.T2055I	Probably damaging	0.999	Non-interacting	Intolerant (0.00)	−0.97	Decrease Stability	−3.05772
p.Y2058C	Probably damaging	1.000	Non-interacting	Intolerant (0.00)	0.27	Increase Stability	−7.73506

AA: amino acid, DDG: delta delta G/free energy change.

## Supplementary Materials

The following are available online at www.mdpi.com/2073-4425/7/11/96/s1. Table S1: PCR primers used for *NSD1* mutational analysis. Table S2: Primers used for RT-qPCR. Table S3: SNPs of *NSD1* found in our 34 Sotos patients.
