# Supplementary material for: Steric Clash in the SET Domain of Histone Methyltransferase NSD1 as a Cause of Sotos Syndrome and Its Genetic Heterogeneity in a Brazilian Cohort
Source: Genes (Basel). 2016 Nov 9;7(11):96. doi: 10.3390/genes7110096 (PMC5126782; doi:10.3390/genes7110096)
Supplement: Supplementary file 1 [file genes-07-00096-s001.docx]

Supplementary Materials: Steric Clash in the
SET Domain of Histone Methyltransferase
NSD1 as a Cause of Sotos Syndrome and Its
Genetic Heterogeneity in a Brazilian Cohort

Kyungsoo Ha, Priya Anand, Jennifer A. Lee, Julie R. Jones, Chong Ae Kim,
Debora Romeo Bertola, Jonathan D. J. Labonne, Lawrence C. Layman,
Wolfgang Wenzel, Hyung-Goo Kim

**Table S1.** PCR primers used for NSD1 mutational analysis.

| **Primer Name** | **PCR Primer Sequence (5′ > 3′)** |
| --- | --- |
| Exon 2.1F | GAGTCAGATGGCCTATTAACTC |
| Exon 2.1R | GAATCTACATCTGCATCGTCC |
| Exon 2.2F | GTAACAAAGACTATCAAGAATGGC |
| Exon 2.2R | GTTTCCCTTTAAGTGGCCTG |
| Exon 3F | AAGGCTAATAGGAATGAC |
| Exon 3R | GTTATTCTAAAGGCAACTGCC |
| Exon 4F | GCTGTTCTCTTAATGATGATGAGAAG |
| Exon 4R | TTCTTACTTGCTTCTGTCACTC |
| Exon 5.1F | CTTCTGATTTCATCTCCCT |
| Exon 5.1R | CTGTGAGGCTATTTGCTATCC |
| Exon 5.2F | TCCAGAGAACCTTGGCCTAAAC |
| Exon 5.2R | TCCAGGCTCTGCACTCTTAG |
| Exon 5.3F | GAAGCCTCTCATTAGTAACTC |
| Exon 5.3R | ATGGCTTTGATGTTCCAGAG |
| Exon 5.4F | ATCCGAGTTGAAGGAACTCTC |
| Exon 5.4R | TGCAGTACAGCATCAAGAGTCAC |
| Exon 5.5F | CTTCATCCAAATTGCGAGATGC |
| Exon 5.5R | CAAGTATGCTTGCTGAAGGAG |
| Exon 5.6F | ACCTCGTAAGCGCATGAACAG |
| Exon 5.6R | CTTCACTTTACCATTACAACAGACC |
| Exon 6F | GGGAGTATCAGATGGTCT |
| Exon 6R | GGACCAGTGAAAGTTTGCTG |
| Exon 7F | ACAATTTTGGCCTGTGGACTC |
| Exon 7R | CATATCTGCTCAAATACTGAGAC |
| Exon 8F | AATTAACTTGTGCCCAGTTTCTAA |
| Exon 8R | CATCAATGTTATAACTGCTACTC |
| Exon 9F | TGGCAGCTGACAATTCAGAC |
| Exon 9R | CTTCAAACTTACTACTGCATTACG |
| Exon 10F | GGACATGTGTGTTAGTAGCCAGC |
| Exon 10R | GGATGTGGCCTCTGGCGTG |
| Exon 11F | GGGTCAAATGGAAGAGACATC |
| Exon 11R | CCATCATAAAGAGATGGAGTGG |
| Exon 12F | TTACTTTAACCCACTGACACTGG |
| Exon 12R | AACTAGCCCAGTGTTGCCAC |
| Exon 13F | CGATGTCAAACCGATCAGTCC |
| Exon 13R | CACAGCGAGACTCTGTCTC |
| Exon 14F | CCATCATCTTAGTGGTCATTCC |
| Exon 14R | CCTGAATGGCAGATGAATAGTATC |
| Exon 15F | CACATACATGACTTGCAGTC |
| Exon 15R | CATTGTATCTGAGAGGTCTC |
| Exon 16F | GCCTTGCAGCCTTCTAGAGG |
| Exon 16R | CAGACACTCAGGTAACAGAGGTC |
| Exon 17F | GAAGTGACTTGTGCTGTCTG |
| Exon 17R | TTCTCTTCTAGAGAAGGTCCC |
| Exon 18F | CGTGAATTGTCTTCTGCTGAC |
| Exon 18R | GATCAATGATATCAAGCAACTGC |
| Exon 19F | TTTGCCATTAAGTCAGGAGG |
| Exon 19R | CCTACAAATACTATGGCTGG |
| Exon 20F | ACAGCAGAGGTCTCAGGAAG |
| Exon 20R | GAAATTCAGATGTCAGCTGCAG |
| Exon 21F | TCTCTTGGGAGTTGGTATCC |
| Exon 21R | CACCACTAATCCCAAAGCAG |
| Exon 22F | GTGTTCACAGAATGCTGACTG |
| Exon 22R | GAGTATGATGGAGAGAACGAG |
| Exon 23.1F | TAGCCTTGGCCCATGTGATATG |
| Exon 23.1R | GCCTCTGACAAGTCCCTGCCAG |
| Exon 23.2F | AGAGCAATCAACAGGAATGGC |
| Exon 23.2R | TGATAGTACTTTCTCAGGAGG |
| Exon 23.3F | ACTTCAGACAGGCCTACTGAC |
| Exon 23.3R | GGCTGAGAAAGAAGTCTGGC |
| Exon 23.4F | GATCCTCTTCAGACATCTGGG |
| Exon 23.4R | GCAGCTTGTTTGTTCATGTGAC |
| * M13F tail | GTAAAACGACGGCCAG |
| * M13R tail | CAGGAAACAGCTATGAC |

Note that large exons were divided into multiple amplicons. * All PCR primers have an M13 tail added, and complementary M13 primers were used for downstream Sanger sequencing.

**Table S2.** Primers used for RT-qPCR.

| **Target** | **Forward Primer (5′–3′)** | **Reverse Primer (5′–3′)** |
| --- | --- | --- |
| NSD1Ex5F2-R2 | TAAAGATGAACGGAGGGGAAAGAT | GTGAGGCTATTTGCTATCCTGGAA |
| NSD1Ex23F2-R2 | GCTGACCCAAGGCTGGATAAATC | GTAATGAGCAGTCTGTCTGGCGG |

**Table S3.** SNPs of *NSD1* found in our 34 Sotos patients.

| **Location** | **Nucleotide Change (NM_022455.4)** | **Amino-Acid Change (NP_071900.2)** | **Frequency in Patients** | **Frequency of Second Allele in Patients** | **dbSNP** |
| --- | --- | --- | --- | --- | --- |
| Exon 5 | c.1482C>T | p.C494C | 18 | 5/68 | rs1363405 |
| Exon 5 | c.1515T>C | p.N505N | 1 | 0/68 | rs114747882 |
| Exon 5 | c.1749G>A | p.E583E | 7 | 0/68 | rs3733874 |
| Exon 5 | c.1840G>T | p.V614L | 7 | 0/68 | rs3733875 |
| Exon 5 | c.2153G>A | p.S718N | 1 | 0/68 | rs781142476 |
| Exon 5 | c.2169C>T | p.T723T | 1 | 0/68 | rs11948062 |
| Exon 5 | c.2176T>C | p.S726P | 7 | 0/68 | rs28932178 |
| Exon 5 | c.3106G>C | p.A1036P | 1 | 0/68 | rs28932179 |
| Exon 5 | c.3307G>A | p.D1103N | 1 | 0/68 | rs746210838 |
| Exon 5 | c.3705T>C | p.N1235N | 8 | 0/68 | rs28932181 |
| Intron 17 | c.5623-22G>A | − | 3 | 0/68 | rs79098301 |
| Exon 23 | c.6750G>A | p.M2250I | 3 | 0/68 | rs35848863 |
| Exon 23 | c.6782T>C | p.M2261T | 3 | 0/68 | rs34165241 |
| Exon 23 | c.6829T>C | p.L2277L | 34 | 26/68 | rs28580074 |
| Exon 23 | c.6903G>C | p.G2301G | 7 | 1/68 | rs11740250 |
| Exon 23 | c.7636G>A | p.A2546T | 2 | 0/68 | rs78247455 |
